# Brain Lipotoxicity of Phytanic Acid and Very Long-chain Fatty Acids. Harmful Cellular/Mitochondrial Activities in Refsum Disease and X-Linked Adrenoleukodystrophy

**DOI:** 10.14336/AD.2015.0823

**Published:** 2016-03-15

**Authors:** Peter Schönfeld, Georg Reiser

**Affiliations:** 1 Institut für Biochemie und Zellbiologie,; 2 Institut für Neurobiochemie (Institut für Inflammation und Neurodegeneration), Medizinische Fakultät der Otto-von-Guericke-Universität Magdeburg, Magdeburg, Germany

**Keywords:** phytanic acid, very long-chain fatty acids (VLCFA), mitochondria, neural cells, peroxisomal disorder, Refsum disease, adrenoleukodystrophy

## Abstract

It is increasingly understood that in the aging brain, especially in the case of patients suffering from neurodegenerative diseases, some fatty acids at pathologically high concentrations exert detrimental activities. To study such activities, we here analyze genetic diseases, which are due to compromised metabolism of specific fatty acids, either the branched-chain phytanic acid or very long-chain fatty acids (VLCFAs). Micromolar concentrations of phytanic acid or of VLCFAs disturb the integrity of neural cells by impairing Ca^2+^ homeostasis, enhancing oxidative stress or de-energizing mitochondria. Finally, these combined harmful activities accelerate cell death. Mitochondria are more severely targeted by phytanic acid than by VLCFAs. The insertion of VLCFAs into the inner membrane distorts the arrangement of membrane constituents and their functional interactions. Phytanic acid exerts specific protonophoric activity, induces reactive oxygen species (ROS) generation, and reduces ATP generation. A clear inhibition of the Na^+^, K^+^-ATPase activity by phytanic acid has also been reported. In addition to the instantaneous effects, a chronic exposure of brain cells to low micromolar concentrations of phytanic acid may produce neuronal damage in Refsum disease by altering epigenetic transcriptional regulation. Myelin-producing oligodendrocytes respond with particular sensitivity to VLCFAs. Deleterious activity of VLCFAs on energy-dependent mitochondrial functions declines with increasing the hydrocarbon chain length (C22:0 > C24:0 > C26:0). In contrast, the reverse sequence holds true for cell death induction by VLCFAs (C22:0 < C24:0 < C26:0). In adrenoleukodystrophy, the uptake of VLCFAs by peroxisomes is impaired by defects of the ABCD1 transporter. Studying mitochondria from ABCD1-deficient and wild-type mice proves that the energy-dependent functions are not altered in the disease model. Thus, a defective ABCD1 apparently exerts no obvious adaptive pressure on mitochondria. Further research has to elucidate the detailed mechanistic basis for the failures causing fatty acid-mediated neurodegeneration and should help to provide possible therapeutic interventions.

## Metabolism of fatty acids at a glance

A characteristic feature of the brain is the high amount of non-triglyceride lipids, which are either membrane components or are involved in signaling pathways. Thus, it becomes increasingly clear that the diversity of cerebral lipids affects a broad range of brain functions, and contributes to or triggers the development of a variety of mental disorders, like depression and anxiety, and neurodegenerative diseases [1, 2]. In addition, mitochondrial dysfunction is considered to be the main causal factor in the pathogenesis of several neurodegenerative diseases (for recent review, see [3]).

Moreover, except for the brain, long-chain fatty acids (LCFAs, fatty acid chain length C12 to C18 carbon atoms) play an important role in the energy metabolism of most tissues. Coenzyme A-derivatives of LCFAs have the highest energy as donors of hydrogen for the oxidative ATP synthesis in cells. However, LCFAs are also involved in the regulation of the energy metabolism of mammals by activation of a group of transcription factors, the peroxisome proliferator activated receptors (PPARs). Recently, we have analyzed the general role of PPARs in the brain [4]. The three PPAR isoforms (α, β/δ and γ) are members of the nuclear receptors superfamily, which are expressed throughout the brain. The main function of nuclear receptors is to regulate gene expression at the level of transcription. This regulation is achieved by the binding of nuclear receptor homodimers or heterodimers to response elements in the promoters of target genes. PPARα induces the activation of genes, which are coding for enzymes of the β-oxidation and the ketogenesis, thereby mediating the metabolic adaptation of the energy metabolism to starvation [4]. In addition, recent data indicate that the PPAR isoforms, PPAR α, β/δ and γ, represent a tightly interconnected array of ligand-activated transcription factors. For this array we coined the term PPAR triad [5, 6]. Synthetic PPARα and PPARγ ligands initiate neuroprotective activity. In this context it is important to note that PPARβ/δ activation emerged as the focus of a novel approach for the treatment of a wide range of neurodegenerative diseases. We propose that PPARβ/δ has a central control of the PPAR triad. Moreover, the PPAR triad concept fits in the view that PPARβ/δ is involved in the regulation of myelination, and glutamate-induced neurotoxicity, and we integrate the PPAR system with signaling of reactive oxygen species/NO/Ca^2+^ pathways [5].

**Figure 1.**
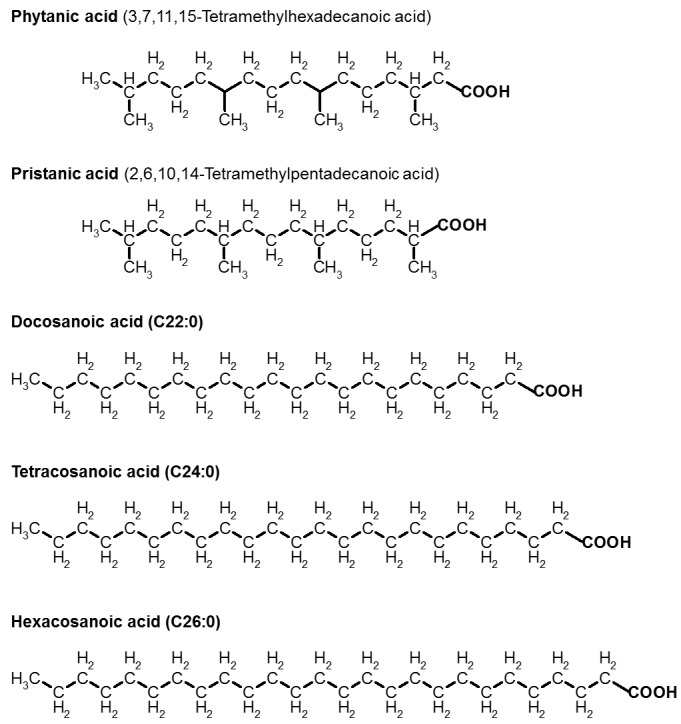
**Structures of phytanic acid, pristanic acid and the VLCFAs**. Shown are docosanoic acid (behenic acid; C22:0), tetracosanoic acid (lignoceric acid; C24:0) and, as described in the text hexacosanoic acid (cerotic acid; C26:0)

The activation of PPARα has been also demonstrated for the branched-chain phytanic acid (see structure in Figure 1) [7-9]. Phytanic acid-induced activation of PPARα is involved in the regulation of carnitine homeostasis and in the induction of various peroxisomal and mitochondrial β-oxidation activities (see [9]). In the skeletal muscle tissue, PPAR β/δ stimulates gene induction for enhancing the oxidation of LCFAs and mitochondrial biogenesis (see for a review [10] and references therein).

In peroxisome-associated diseases, such as adrenoleukodystrophy or Refsum disease, an age-dependent accumulation of very long-chain fatty acids (VLCFAs; fatty acid chain length ≥22 carbon atoms) or branched-chain LCFAs takes place in several tissues, respectively [11, 12]. Moreover, accumulation of VLCFAs in cortical brain regions is also documented in Alzheimer’s disease patients [13].

Generally, supraphysiological tissue concentrations of non-esterified LCFAs impair the mitochondrial physiology (see for a review [14] and references therein). Briefly, free LCFAs partly impair ATP regeneration, depolarize mitochondria, sensitize mitochondria to undergo permeability transition and stimulate mitochondria-associated as well as non-mitochondrial oxidative stress (see for a review [15] and references therein). Distinct proteins have been found to contribute to the mechanism of permeability transition pore opening (for review see [16] and references therein). Despite the ongoing intensive research on the protein composition of the permeability transition pore (PTP), its composition appears still enigmatic [17].

In sharp contrast to the situation in other organs with high energy demand, such as liver, heart, skeletal muscle, or kidney, LCFAs play only a minor role as hydrogen source in the energy metabolism of the brain tissue [18, 19]. This well-known paradigm is substantiated by the two following findings. Firstly, brain mitochondria have only poor enzymatic capacity for β-oxidation of fatty acids [20]. Secondly, prior to β-oxidation in the mitochondrial matrix, free LCFAs have to be converted into CoA-thioesters and, thereafter, translocated as acyl-carnitines across the inner membrane (IM) of mitochondria. Conversion of CoA-thioesters into the carnitine-derivatives is catalyzed by the carnitine palmitoyltransferase I (CPT I), the rate-limiting step of β-oxidation. Surprisingly, the brain-specific CPT Ic is unable to convert fatty acid acyl-CoAs to fatty acid acyl-carnitines [21, 22].

However, fatty acids supply also brain mitochondria with reducing equivalents as ketone bodies, which are generated from fatty acids in the liver during extended periods of starvation. It has to be noted, however, that in the cerebral tissue, glucose degradation and the subsequent oxidation of pyruvate is the main source for supplying hydrogen to the machinery of oxidative ATP generation.

In contrast to LCFAs, mitochondria are not able to degrade the long branched-chain phytanic acid and VLCFAs (fatty acid chain length ≥22 carbon atoms). The structures of the C22:0, C24:0 and C26:0, as well as of phytanic acid and pristanic acid fatty acids are displayed in Figure 1. Phytanic acid and VLCFAs are partly degraded by the peroxisomal β-oxidation [23]. The degradation products, such as medium-chain fatty acid-CoAs, propionyl-CoA and acetyl-CoA, are released to mitochondria, after conversion into acyl-carnitines or non-esterifies short-chain fatty acids (see for a review [24]).

Peroxisomes are essential organelles of the cytosol in higher eukaryotic cells with multiple metabolic functions, including the peroxisomal pathway of the β-oxidation [25, 26].

Consequently, enzymatic defects of the peroxisomal β-oxidation or of the uptake of fatty acids into peroxisomes results in enhanced serum and tissue levels of several fatty acids. Most prominent are in this context the phytanic acid and VLCFAs (see for a review [27] and references therein). Enhanced tissue levels of VLCFAs promote their esterification with cholesterol, phospholipids and gangliosides and further, the formation of lipid-containing inclusion bodies within certain cells, such as adrenocortical cells, Schwann cells or brain macrophages (see reviews [11, 28] and references therein). In addition, peroxisomal β-oxidation of fatty acids is also a source of the generation of reactive oxygen species (ROS), in particular H_2_O_2_ [26].

Elevated serum levels of phytanic acid and of VLCFAs are the characteristic biochemical hallmarks of two inherited neurodegenerative diseases, firstly, the adult form of the Refsum disease [12] and, secondly, the X-linked adrenoleukodystrophy (X-ALD) [29].

The finding that both diseases are paralleled by tissue lipid-storage of phytanic acid or that of VLCFAs, respectively, led to the hypothesis that an impaired cerebral metabolism of phytanic acid and that of VLCFAs, respectively, is the cause of the pathogenesis of these neurodegenerative diseases. Moreover, based on the well-known susceptibility of the mitochondrial physiology to LCFAs, it is most likely that mitochondria become also targeted by phytanic acid and VLCFAs. This view further leads to the idea that an impaired cerebral energy metabolism is a causal risk factor of the pathogenesis of Refsum disease and the X-ALD. Besides the impaired oxidative ATP synthesis, fatty acid-induced mitochondria-associated oxidative stress is considered to be a further detrimental factor of pathomechanisms (see for recent reviews [30-32] and references therein).

The aim of our review is to summarize the results obtained in recent experimental work on harmful activities of the branched-chain phytanic acid as well as that of VLCFAs on neural cells and further, on isolated brain mitochondria for a deep understanding of mechanistic details.

## Degradation of phytanic acid and very long-chain fatty acids

Phytanic acid (3,7,11,15-tetramethylhexadecanoic acid), a saturated branched-chain fatty acid of 20 C atoms with a poly-isoprenoic structure, is formed by bacterial activities from the phytol side chain of chlorophyll in the stomach of ruminants (for review see [27]). Humans take up phytanic acid in the esterified form, mostly in red meat (cow and sheep) and milk products [33]. Due to the methyl group in the *β*-position, phytanic acid is not directly amenable to *β*-oxidation. Phytanic acid is degraded in mammalian cells by combined peroxisomal and mitochondrial *β*-oxidation [27]. After the uptake of phytanic acid from the blood, the activation to phytanoyl-CoA can occur at three sites, mitochondria, endoplasmic reticulum, and peroxisomes. Briefly, the degradation of phytanoyl-CoA inside the peroxisomes starts with shortening by one carbon atom via the *α*-oxidation process to pristanic acid (2,6,10,14-tetramethylpentadecanoic acid), which is also displayed in Figure 1. In humans and in rats, *α*-oxidation is catalyzed by phytanoyl-CoA 2-hydroxylase, thereby producing *α*-hydroxyphytanoyl-CoA. Thereafter, 2-hydroxy-3-methylacyl-CoA lyase cleaves *α*-hydroxyphytanoyl-CoA into pristanal and formyl-CoA. Oxidation of pristanal generates pristanic acid (see Figure 1), which after activation to the CoA derivative is degraded by normal peroxisomal *β*-oxidation to 4,8-dimethylnonanoyl-CoA. Before *β*-oxidation starts, (2*R*)-stereoisomers of 2-methyl-branched fatty acyl-CoAs are converted into the (2*S*)-stereoisomers by *α*-methylacyl-CoA racemase, whereas the *β*-oxidation pathway accepts only this stereoisomer. Furthermore, 4,8-dimethylnonanoyl-CoA is exported to mitochondria as a carnitine derivative, where it is further oxidized by the mitochondrial *β*-oxidation. In summary, one molecule of pristanoyl-CoA is degraded to three molecules of acetyl-CoA, three molecules of propionyl-CoA and one molecule of isobutyryl-CoA.

In the Refsum disease, phytanic acid accumulates throughout the body as a consequence of mutations in two genes, PHYH (the gene encoding the phytanoyl-CoA hydroxylase and PEX7 (encoding the PTS2 receptor) [34]. In patients suffering from this disease, the total plasma concentration of phytanic acid may exceed 5,600 μM with a control range of 0 - 9 µM [34]. Clinical features of Refsum disease, such as cardiac malfunctions and those in the olfactory and auditory nerves, suggest that the supraphysiological concentration of phytanic acid exerts cytotoxic activities, which are most prominent in tissues with a high oxidative ATP generation, such as brain and heart [34].

Biochemistry of phytanic acid differs from that of its unbranched homologue, palmitic acid. Thus, intracellular fatty acid-binding proteins promote to a lesser extent the esterification and oxidation of phytanic acid, as compared to that of palmitic acid [35]. This implies that non-esterified phytanic acid may increasingly accumulate to high intracellular levels enhancing its potential cytotoxicity [36]. Furthermore, incorporation of esterified phytanic acid into membranes will distort the arrangement of membrane constituents and their functional interactions due to its bulky hydrocarbon tail [37, 38]. Alteration of the organization of the IM by phytanic acid has been shown by ESR spectroscopy [39] using the lipid-specific spin probe 5-doxylstearic acid (5-DSA) and the protein-specific spin probe MAL-TEMPO (4-maleimido-2,2,6,6-tetramethyl-piperidine-1-oxyl). These studies showed that phytanic acid (i) increased the mobility of phospholipid molecules and (ii) altered the conformational state and/or the segmental mobility of membrane proteins. Generally, insertion of phytanic acid into the IM alters membrane properties more than the straight-chain analogue palmitic acid.

Saturated VLCFAs are found in mammals as constituents of sphingolipids, particularly in those in the outer leaflet of the plasma membrane. VLCFAs become absorbed from the diet. On the other side, VLCFAs that accumulate in X-ALD are mostly formed by an induced endogenous elongation of LCFAs, a process mostly localized in the endoplasmic reticulum [11, 40]. VLCFAs are activated in the cytosol by the VLCFA acyl-CoA synthase to CoA-thioesters. Mammalian peroxisomes are equipped with three ABC transporters (ABCD1-3), which shuttle in an ATP-dependent manner a distinct spectrum of fatty acyl-CoA-thioesters (including VLCFAs and others, such as phytanic acid, arachidic acid or ω-9 monosaturated fatty acids) across the peroxisomal membrane [25]. Thereby, VLCFAs were transported mostly by the ABCD1, since in case of an ABCD1 deficiency the expression of ABCD2 and ABCD3 can only partly compensate for the reduced uptake of VLCFAs [41].

Similar to phytanic acid, activated VLCFAs are degraded by cooperation of the peroxisomal and mitochondrial β-oxidation pathways [11]. This combined degradation of VLCFAs by peroxisomes and mitochondria is a typical example for the concept, according to which the relationship between peroxisomal and mitochondrial metabolism has been described in a picturesque manner as the relationship between the little sister and the big brother [42].

VLCFAs are extremely hydrophobic and they have physiological properties different from those of LCFAs. The rate of desorption from biological membranes decreases exponentially with increasing chain length. Desorption of C26:0 from a lipid membrane is 10,000 times slower than that of C16:0 and C18:0 fatty acids [43]. Moreover, it has been shown that the saturated hexacosanoic acid (C26:0) binds to phospholipid vesicles and exhibits an ionization behavior in the membrane which is indistinguishable from that of other fatty acids. Similar to phytanic acid, membrane-incorporated VLCFAs perturbate the surrounding phospholipids in a manner distinct from LCFAs. Thus, enrichment of VLCFAs in membrane glycerolipids caused dramatic alterations in plant morphology [44].

**Figure 2.**
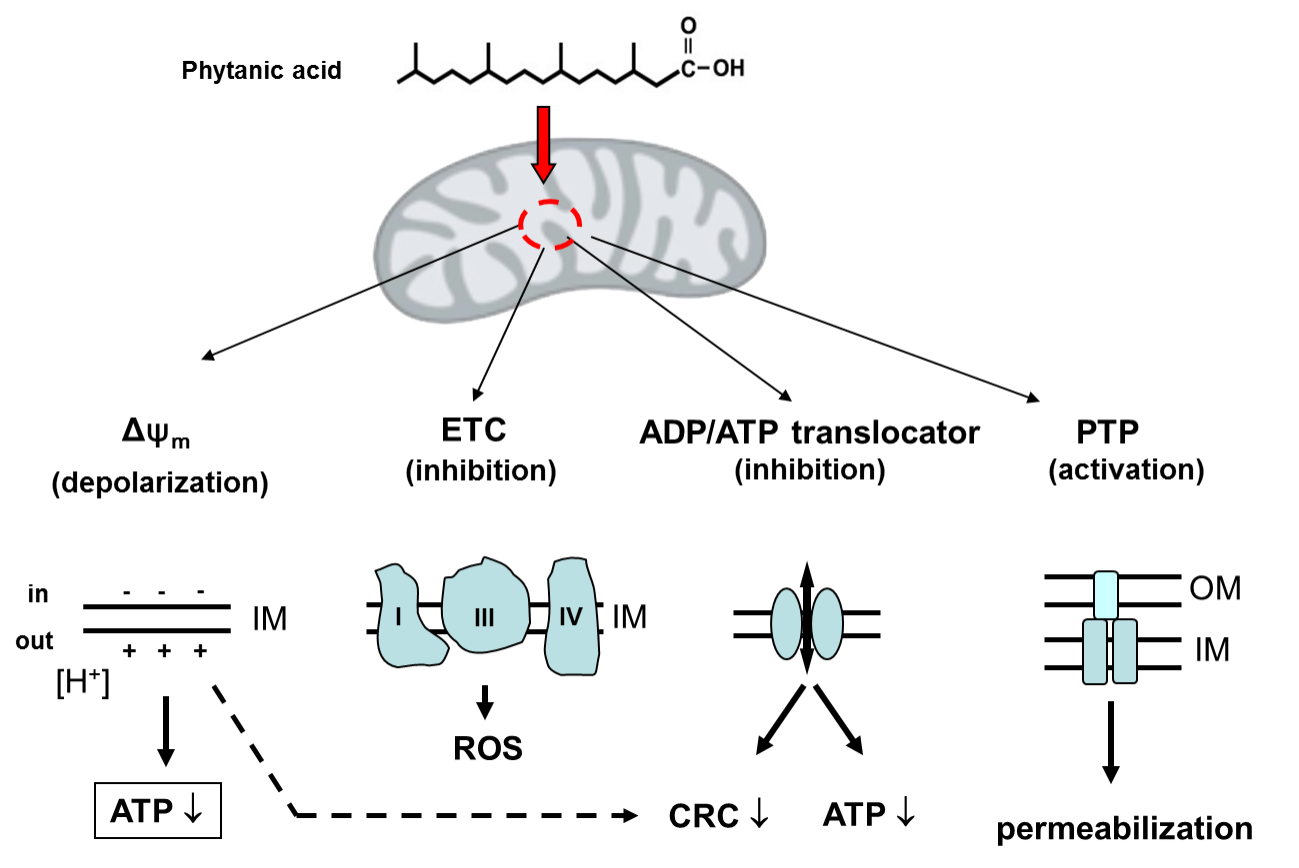
**Phytanic acid impairs mitochondrial energy-dependent functions**. Acute effects: Phytanic acid impairs the mitochondrial physiology. As protonophore, phytanic acid partly decreases Δψ_m_ at the inner membrane (IM). This depolarization uncouples the oxidative phosphorylation, thereby reducing ATP synthesis. Binding of phytanic acid to the electron transport chain (ETC) complexes interferes with the electron transport, thereby stimulating the generation of ROS as byproduct of ETC activity. Binding of phytanic acid to the ADP/ATP translocator partly inactivates the exchange of ADP and ATP across the IM and fixes the ADP/ATP carrier in a conformational orientation within IM, where the binding site for adenine nucleotides faces the cytosol (c-state). Both, phytanic acid-induced decrease of Δψ_m_ and phytanic acid binding to the ADP/ATP translocator decrease the Ca^2+^ retention capacity (CRC) and sensitize the permeability transition pore (PTP) to open, thereby releasing mitochondrial proapoptotic factors (e.g., cytochrome c, AIF, Smac-Diablo).

## Alteration of energy-dependent functions of mitochondria and brain cells by phytanic acid

### 1. Mitochondrial uncoupling, Ca^2+^ retention and permeability transition

It has been observed that the exposure of isolated mitochondria to micromolar concentrations of phytanic acids impairs the integrity of the IM and several, membrane-linked energy-dependent functions, which are shown in a simplified form in Figure 2. The perturbation of the arrangement of membrane constituents by incorporation of phytanic acid into the IM is likely to enhance the passive permeability of the IM to H^+^ [38]. Besides the induction of a membrane leakage to H^+^ due to membrane distortion, phytanic acid also exerts protonophoric activity. In comparison to the straight-chain palmitic acid, phytanic acid is a stronger protonophore. Thus, the phytanic acid-linked depolarization of the energized IM can be attributed to an increased permeability of the IM to H^+^ resulting from membrane distortion and protonophoric activity [45-47]. The protonophoric activity of phytanic acid is attributed to initiation of fatty acid cycling across the IM. This process consists of the membrane permeation by the protonated form of fatty acids and, the back-transport of their anionic form by the ADP/ATP-translocase and, probably, other integral membrane proteins in the IM [48, 49]. Consequently, phytanic acid uncouples the F_O_F_1_-ATPsynthase reaction from the electron transport within the respiratory chain, thereby reducing oxidative ATP generation. Furthermore, phytanic acid binds to the ADP/ATP-translocase [46], thereby causing a moderate inhibition of the ADP/ATP exchange across the IM [45].

Protonophoric uncoupling by phytanic acid has also been demonstrated with human skin fibroblasts [50]. This study revealed that an enrichment of phytanic acid in cells decreases the mitochondrial membrane potential (Δψ_m_), the NAD(P)H autofluorescence and, thereby, the ATP synthesis.

Moreover, brain mitochondria respond to low micromolar concentrations of phytanic acid with a dramatic decline of their Ca^2+^ retention capacity (CRC). Such decline of CRC was not found with the methylester of phytanic acid [51]. Since Ca^2+^ uptake by energized mitochondria is driven by the Δψ_m_ at the IM, and, in addition, esterified phytanic acid is unable to depolarize mitochondria, it is tempting to speculate that a lowered Δψ_m_ is causal for the diminished CRC. Otherwise, slight depolarization by chemical uncouplers, like FCCP, is nearly without any effect on the CRC of energized mitochondria. Thus, it can be hypothesized that the interaction of phytanic acid with the ADP/ATP-translocase contributes to the reduction of CRC (see Figure 2). Such explanation is consistent with the well-established finding that binding of certain ligands to the ADP/ATP-translocase (e.g., by carboxyatractyloside, bongkrekic acid, acyl-CoA) affects the tendency of mitochondria to undergo permeability transition [52, 53].

Important for functional consequences is the finding that mitochondria, which are preloaded with small amounts of Ca^2+^ (100 nmol/mg of protein), became highly sensitized to rapid permeability transition even when only relatively low concentrations of phytanic acid (below 5 μM) were applied [45, 46] (see Figure 2). In conclusion, in the neural tissue, which becomes enriched with phytanic acid, the reduction in mitochondrial ATP regeneration and the facilitation of the opening of the PTP are two major mechanisms by which the branched-chain fatty acid phytanic acid induces the onset of degenerative processes. Taken together, elevated concentrations of phytanic acid exert multiple direct disturbances of energy-dependent mitochondrial functions (see Figure 2.).

### 2. ROS generation and oxidative stress

In addition, low concentrations of phytanic acid impaired the electron transport in the respiratory chain. The interference of phytanic acid with the electron transport was demonstrated by inhibition of FCCP-uncoupled respiration, the inactivation of the NADH-ubiquinone oxidoreductase complex in permeabilized mitochondria, the decrease in the reduction of the synthetic electron acceptor 3-(4,5-dimethylthiazol-2-yl)-2,5-diphenyl-tetrazolium bromide (MTT) in resting mitochondria, and the increase of the mitochondrial NAD(P)H level in FCCP-uncoupled mitochondria [30, 45, 54].

As consequence of slowing down the impaired electron transport by phytanic acid, the mitochondrial ROS generation becomes increased [54, 55]. This is clearly demonstrated when mitochondria isolated from brain or heart of adult rats were expose to low micromolar concentrations of phytanic acid. In addition, phytanic acid has higher potency than palmitic acid to enhance the mitochondrial ROS generation [51, 54, 56]. Generally, fatty acid-induced stimulation of the ROS generation has been attributed to impaired forward electron transport in the electron transport chain [55], where the electron transport from complex I to complex IV becomes partly blocked. In this condition, the leakage of electrons, mostly from complex I and III to molecular O_2_ becomes facilitated, resulting finally in an enhanced generation of ROS (see Figure 2).

Moreover, the exposure of mitochondria to phytanic acid enhanced oxidative stress inside the mitochondria, indicated by an inactivation of the superoxide-sensitive aconitase (an enzyme of the citric acid cycle), the partly oxidized matrix glutathione pool, enhanced lipid peroxidation and protein carbonyl formation [30, 54, 55].

Finally, in line with studies on isolated brain mitochondria, it has been demonstrated that phytanic acid enhanced the ROS generation inside of neural cells [57, 58]. These results were obtained using dihydroethidium, a fluorescence-probe detecting superoxide in the cytosolic compartment.

### 3. Cellular Ca^2+^ handling and apoptotic cell death

For getting deeper insights into the pathogenesis of the Refsum disease, the influence of phytanic acid on cellular physiology of rat hippocampal astrocytes was examined [57]. The first question was: Does phytanic acid initiate mitochondrial depolarization *in situ*? The Δψ_m_ was determined *in situ* by the cationic fluorescent probe Rh123. In astrocytes, which were incubated with Rh123, the energized mitochondria accumulate the dye within the matrix. The cells were then superfused with 100 µM of phytanic acid or palmitic acid as fatty acids for 10 min. Thereafter, mitochondria were fully uncoupled by addition of FCCP (4 µM) and oligomycin (Oli; 10 µM) to the perfusion medium. With this experimental strategy it was found that phytanic acid evoked a steady increase in the fluorescence signal due to release of accumulated Rh123, clearly indicating the decrease of the Δψ_m_. Palmitic acid, however, had no comparable effect on the Δψ_m_.

Since membrane-incorporated phytanic acid changed the membrane structure (see above), there is good reason to examine a possible effect of phytanic acid on the permeation of the astrocytic plasma membrane to extracellular Ca^2+^. For examination, cytosolic Ca^2+^ concentration in hippocampal astrocytes was measured by fura-2 fluorescence. In summary, exposure of astrocytes to 100 µM phytanic acid evoked an immediate increase in the cytosolic Ca^2+^ concentration [57]. In contrast, the application of phytanic acid in EGTA-containing Ca^2+^-free medium resulted only in a transient Ca^2+^ peak, indicating the release of Ca^2+^ from intracellular stores. Despite the continued presence of phytanic acid, the cytosolic Ca^2+^ concentration completely recovered. In contrast, palmitic acid elicited only a negigible increase of the cytosolic Ca^2+^ concentration. Thus phytanic acid exerts a specific influence on hippocampal astrocytes, which is generally not detectable with straight-chain, saturated LCFAs.

Pristanic acid, a homologue of phytanic acid (see Figure 1), is formed by shortening of phytanic acid by one C atom via the *α*-oxidation process. Similar to phytanic acid, pristanic acid exerts a strong cytotoxic activity on brain cells, displayed by dramatic Ca^2+^ deregulation, *in situ* mitochondrial depolarization and cell death [58]. With respect to phytanic acid, pristanic acid strongly induced the generation of ROS, whereas phytanic acid exerts weaker effects on ROS production. Thus, it can be summarized, that pristanic acid as well as phytanic acid induced a complex array of toxic activities with mitochondrial dysfunction and Ca^2+^ deregulation, such as the InsP_3_-Ca^2+^ signaling pathway in glial cells.

### 4. Cell signaling: activation of the plasma membrane receptor GPR40

Activation of an intracellular Ca^2+^ signaling pathway by phytanic acid and pristanic acid suggests that a membrane receptor coupled to intracellular Ca^2+^ release might be involved. The identification of this receptor might contribute to the understanding of the phytanic acid- and pristanic acid-mediated toxicity. A receptor candidate is the free fatty acid receptor GPR40 (also known as FFAR1). This G protein-coupled receptor has been described to be activated by medium- and long-chain saturated and unsaturated fatty acids [59, 60]. The GPR40 receptor leads to activation of phospholipase C (PLC) and intracellular Ca^2+^ mobilization from the endoplasmic reticulum via the inositol 1,4,5-trisphosphate pathway [61, 62]. Besides the medium- and long-chain saturated and unsaturated fatty acids, GPR40 is activated by a wide range of novel agonists, e.g. drugs used in the treatment of diabetes mellitus type 2 (the thiazolidinediones rosiglitazone and troglitazone), the branched-chain dicarboxylic acid MEDICA16 (3,3,14,14-Tetramethylhexadecanedioic acid) or the selective synthetic agonist GW9508 (see [63], and references therein). Moreover, we could demonstrate that phytanic acid and pristanic acid were able to activate the receptor GPR40, which recognizes free fatty acids [63]. Furthermore, we have obtained evidence that the GPR40 activation might be due to an interaction of the carboxylate moiety of fatty acids with the receptor. In summary, our findings suggest also that the phytanic acid- and pristanic acid-mediated Ca^2+^ deregulation involve the activation of GPR40.

## Very long-chain fatty acids and neural cells

### 1. Effects of very long-chain fatty acids on astrocytes, oligodendrocytes and neurons

X-ALD is a severe neurodegenerative disorder resulting from defective ABCD1 transporter protein [64]. ABCD1 mediates peroxisomal uptake of free VLCFAs as well as their CoA-esters. Consequently, VLCFAs accumulate in patients' plasma and tissues, which is considered as pathogenic X-ALD-triggering factor (see for review [11]). Clinical symptoms are mostly manifested in neural tissues and adrenal gland.

Previous studies analyzed the development of X-ALD in humans and gene knockout animal models. However, the toxic effect of VLCFAs leading to severe symptoms with progressive and multifocal demyelination, adrenal insufficiency and inflammation still remains unclear. To understand the toxic effects of VLCFAs in the brain, neural cells were exposed to VLCFAs, and cellular consequences were analyzed [65]. Thereby, it was found that oligodendrocytes and astrocytes challenged with docosanoic (C22:0), tetracosanoic (C24:0) and hexacosanoic acids (C26:0) undergo cell death within 24 h (see structures in Figure 1). VLCFAs-induced depolarization of the IM of mitochondria *in situ* and increased intracellular Ca^2+^ level in all three brain cell types provide indications about the mechanism of toxicity of VLCFAs.

Oligodendrocytes support the myelination of axons in the brain, thereby enabling a rapid propagation of the action potential along axons (for a recent review see [66]). Thus, it is clear that a perturbation of the myelination process impairs essential neuronal functions. Interestingly, VLCFAs affect to a very large degree the myelin-producing oligodendrocytes. In isolated mitochondria, VLCFAs exert detrimental effects by affecting the IM and promoting the permeability transition. In conclusion, we suggest that there is a potent toxic activity of VLCFAs due to dramatic cell physiological effects with mitochondrial dysfunction and Ca^2+^ deregulation. This provides the first evidence for mitochondrial-based cell death mechanisms in neurodegenerative disease with peroxisomal defects and subsequent VLCFAs accumulation.

For further elucidation of the VLCFAs-triggered pathogenesis of X-ALD, astrocytes from wild-type control and a genetic X-ALD mouse model (Abcd1-knockout) were exposed to supraphysiological concentrations of VLCFAs (C22:0, C24:0 and C26:0) [67].

**Figure 3.**
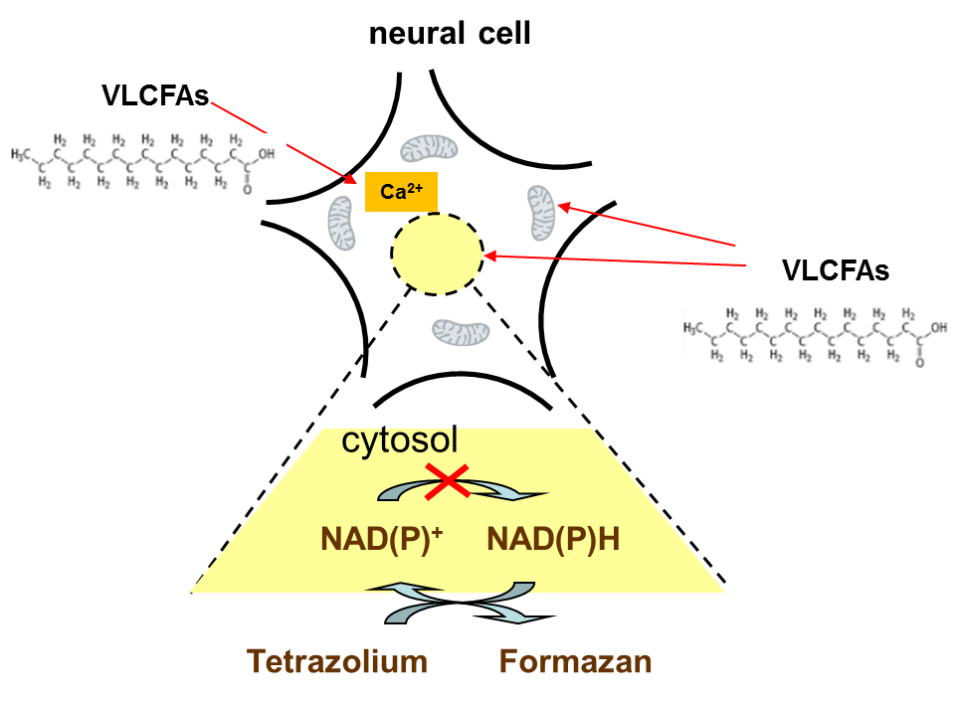
**VLCFAs impair the reduction of oxidized pyridine nucleotides in neural cells**. A marked feature of effects of VLCFAs on the cellular energy metabolism is inhibition of the reduction of the tetrazolium electron acceptor (WST-1) to the formazan dye, which is detected mostly in ABCD1^-/-^ cells. This finding strongly suggests that an enhanced level of VLCFAs diminishes the capacity of Abcd1^-/-^ astrocytes to revert oxidized pyridine nucleotides to NAD(P)H. In addition, VLCFAs impair the cellular Ca^2+^ homeostasis, whereas, in contrast, the mitochondrial Ca^2+^ retention capacity is not affected. Moreover, VLCFAs impair the mitochondrial physiology, thereby partly depolarizing mitochondria and stimulating electron transport chain-associated ROS generation (the latter activities are not shown).

Thereby it becomes evident that VLCFAs exhibit multiple impairments of energy metabolism. For illustration, long-term application of VLCFAs induces high ROS generation, and a strong *in situ* depolarization of mitochondria in Abcd1^-/-^ astrocytes. High ROS generation in VLCFAs-treated astrocytes were found, especially in Abcd1^-/-^ astrocytes after exposure to C26:0, a VLCFA, which is highly increased in X-ALD patients. This indicates an increased vulnerability of Abcd1^-/-^ cells to C26:0 and a crucial role of C26:0 in the pathogenesis of X-ALD.

However, most surprising was the observation that in VLCFAs-exposed Abcd1^-/-^ astrocytes, the reduction of the tetrazolium electron acceptor (WST-1) to the formazan dye was severely diminished. WST-1 is a cell-impermeable, water-soluble electron acceptor, which becomes reduced by electrons derived from the catabolic cell metabolism. These electrons were transmitted from the cytosolic to the extracellular compartment across the cell plasma membrane by 1-methoxy phenazine methosulphate, an artificial membrane-permeable “electron transporter”. Therefore, reduction of extracellular WST-1 strongly suggests that an enhanced level of VLCFAs diminished the capability of Abcd1^-/-^ astrocytes to revert oxidized pyridine nucleotides to NAD(P)H (Figure 3).

In summary, VLCFAs increase the vulnerability of Abcd1^-/-^ astrocytes more than that of astrocytes from wild-type mice. In addition, the observed differences in responses of mitochondria and astrocytes to VLCFAs with the different hydrocarbon chain length suggest that detrimental VLCFAs activities in astrocytes involve defective cellular functions other than mitochondria.

### 2. Comparison of brain mitochondria from wild-type and ABCD1^-/-^ mice

Degradation of VLCFAs is orchestrated by the peroxisomal and mitochondrial β-oxidation pathways (see for review [23]). This combined degradation by both organelles is disturbed in the X-ALD genetic model. From this fact the important question arises: Does the biogenesis of mitochondria respond to the defective ABCD1 protein, leading to mitochondrial functions in the ABCD1-deficient phenotype that are different from that of the normal phenotype?

Indeed, there are the following controversial reports on alterations of mitochondrial functions in adrenoleukodystrophy. On the one side, structural abnormalities of mitochondria in cells of X-ALD mice have been proposed to indicate impaired mitochondrial functions [68, 69], whereas on the other side it was reported that normality in size, structure and localization of mitochondria in muscle can be detected in an ABCD1-deficient mouse model for X-ALD [70]. Rates of oxygen uptake of phosphorylating isolated skeletal muscle mitochondria from ABCD1-deficient and wild-type mice also did not differ [70]. In contrast, experiments with permeabilized spinal cord slices of the ABCD1-deficient mouse model revealed a decrease of the phosphorylating respiration by 20 to 25% [71]. This decrease in the phosphorylating respiration might be attributed to an oxidative impairment of the F_O_F_1_-ATP synthase activity [70] and/or to the noxious activity of an enhanced level of VLCFAs in the spinal cord slice tissue. The latter view is indicated by the observation that the oligomycin-sensitive respiration of fibroblast cultures prepared from control ABCD1-deficient mice (metabolizing galactose) was similar, but decreased in the presence of 50 μM of C26:0 [71]. In addition, reduced contents of mitochondria have been reported in neural tissues from the spinal cords of the same mouse model [72].

The following results were found in our studies focused on brain mitochondria of wild-type and ABCD1-deficient mice [67]. Notable is our finding that the energy-dependent parameters (Δψ_m_, ADP-dependent respiration and ROS generation) in untreated brain mitochondria either measured *in situ* or *in vitro* do not differ for wild-type and ABCD1^-/-^ mice. This suggests that the loss of the ABCD1 protein from peroxisomes has no direct effect on these functional mitochondrial parameters. This is also in line with our observation that changes of the Δψ_m_ and those of the ROS production are comparable in untreated astrocytes from wild-type and ABCD1-deficient mice.

Furthermore, brain mitochondria from Abcd1^-/-^ mice and wild-type control respond similarly to VLCFAs with increased ROS generation, impaired oxidative ATP synthesis and diminished Ca^2+^ uptake capacity, suggesting that a defective ABCD1 exerts no adaptive pressure on mitochondria.

## Perspectives

**Figure 4.**
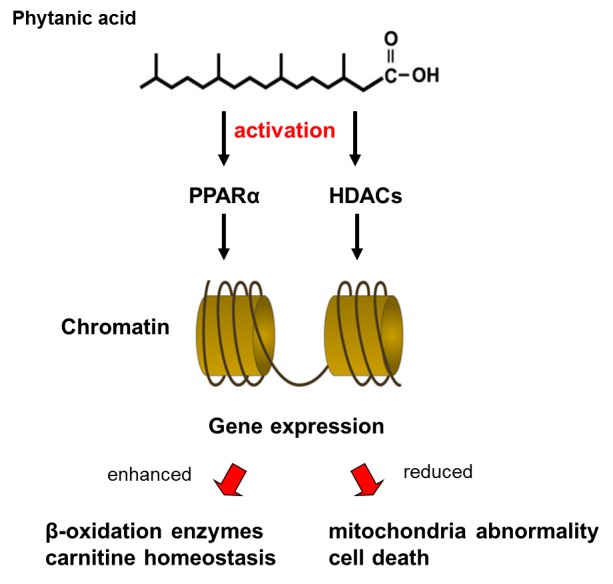
**Phytanic acid alters transcriptional activities of the genome**. Long-term effects: Phytanic acid activates peroxisome proliferation-activated receptor (PPARα) and histone deacetylases (HDACs). Activation of PPARα by phytanic acid induces mostly the expression of enzymes of the peroxisomal β-oxidation pathway and contributes to the carnitine homeostasis. Activation of HDACs reduces histone acetylation, thereby preventing the action of transcription factors. Consequently, mitochondrial abnormalities may develop, which finally contribute to accelerated cell death.

Low micromolar concentrations of phytanic acid or of VLCFAs disturb the integrity of neural cells by impairing the Ca^2+^ homeostasis, enhancing oxidative stress or de-energizing mitochondria. Finally, the combined operation of these harmful activities results in an accelerated cell death. Mitochondria are more severely targeted by phytanic acid. Phytanic acid exerts high protonophoric activity; it is a stronger inducer of ROS generation, partly inactivates the ADP/ATP translocase and reduces ATP generation. A marked inhibition of the Na^+^, K^+^- ATPase activity has also been reported [73]. Despite direct activities on the cellular metabolism, phytanic acid may produce neuronal damage in Refsum disease by an epigenetic transcriptional activity. Using Neura2a cells, it has been demonstrated that even such low micromolar concentrations of phytanic acid as 5 µM, enhance histone deacetylase activity [74]. Consequently, the level of acetylated lysine residues on histones declines, a situation which changed the histone structure and thus, the transcription factors become inactivated. Furthermore, it has been hypothesized, that the reduction of histone acetylation in Neuro2a cells results in mitochondrial abnormalities, which, finally, contribute to an accelerated cell death in Refsum disease (Figure 4).

Myelin-producing oligodendrocytes respond particularly sensitively to the exposure to VLCFAs. However, the deleterious activity of VLCFAs on energy-dependent mitochondrial functions declines with increasing hydrocarbon chain length (C22:0 > C24:0 > C26:0). In contrast, the reverse sequence is increasingly inducing cell death by VLCFAs and reduction of the tetrazolium salt (WST-1). In this case, hexacosanoic acid (C26:0) operates with the highest detrimental potency.

Studying mitochondria from ABCD1-deficient and wild-type mice suggests that their energy-dependent functions are comparable. Thus, a defective ABCD1 exerts obviously no adaptive pressure on mitochondria. This conclusion, however, contradicts a recent study, where the ABCD1 protein was silenced in B12 oligodendrocytes and U87 astrocytes [75]. *In situ* measurements of mitochondrial properties have shown that ABCD1 silencing resulted in reduced activities of electron transport chain-related enzymes and of citric acid cycle. In addition, the mitochondrial redox status was dysregulated and the Δψ_m_ was disrupted following ABCD1 silencing. To date, the reason for this discrepancy between these findings and ours as well as others is not clear.

As specific therapeutic outlook we propose: A disturbed Ca^2+^ signaling seems to be involved in the pathogenesis of X-ALD. Thus, Abcd1^-/-^ astrocytes displayed a much lower intracellular Ca^2+^ reaction in response to acute VLCFAs application and a higher uptake of Ca^2+^ by mitochondria, a finding which contrasts with the observed lack in difference in mitochondrial functions in control mitochondria and in Abcd1^-/-^ deficient mitochondria.

Ca^2+^, which plays a key role in neural signaling in the brain, is of paramount importance also for the control of neurodegenerative diseases, like Alzheimer’s disease, Parkinson’s disease, and Huntington’s disease [76]. Thus, the weaker Ca^2+^ response to VLCFAs in Abcd1^-/-^ astrocytes highlights a further physiological parameter and indicates a therapeutic target for X-ALD.
